# Origin, Impact, and Solutions for Lifestyle-Related Diseases in Japan

**DOI:** 10.7759/cureus.90184

**Published:** 2025-08-15

**Authors:** David Cai, Mohsin Yakub

**Affiliations:** 1 Medicine, California University of Science and Medicine, Colton, USA; 2 Medical Education/Physiology, Nutrition, California University of Science and Medicine, Colton, USA

**Keywords:** chronic disease, hypertension, japan, lifestyle medicine, nutrition, obesity

## Abstract

Japan is an archipelago west of the Pacific Ocean, facing decades of dietary and lifestyle westernization and its physiological consequences. Much like many other countries, Japan is tackling the global trend of lifestyle-related problems. This paper aims to discuss the current demographics in Japan and examine the origin and impact of lifestyle-related chronic diseases within the population. This review will highlight the nutritional transition during the country's development and examine the pathogenesis and pathophysiology of lifestyle-related diseases, primarily hypertension and obesity, in the context of traditional practices and cultural shifts. A combination of high salt intake in traditional cuisine and increased fat and sugar consumption from the westernization of the diet contributes to elevated blood pressures and obesity rates in Japan. This paper explores policy-based interventions, such as nutrient taxation and front-of-package labeling (FOPL), and their potential to mitigate these growing health concerns. One strategy is a nutrient tax to reduce the appeal of specific unhealthy products, such as sugar-sweetened beverages. Similarly, targeting alcohol with taxes and stricter regulations on advertising can reduce consumption. FOPL provides clear and concise nutritional information on the front of each product for consumers to make informed purchases. Expansion of blood pressure monitoring can allow patients to take a more proactive role in their own health, and providers can better tailor treatment plans. Increasing physical activity through workplace wellness programs can reduce the risk of chronic diseases and can be especially effective for Japanese office workers who tend to spend a lot of time in sedentary behavior. Collaboration amongst the government, businesses, and healthcare providers will be vital to empowering the general population to make healthy changes.

## Introduction and background

The prevalence and rising incidence of lifestyle-driven chronic diseases are affecting much of the world, contributing to a large majority of deaths worldwide and posing a monumental burden on individuals and the health care system [1]. Chronic diseases, such as cardiovascular disease and obesity, were not always so common, but incidence and prevalence have been increasing in every region and across all socioeconomic classes. To understand how and why Japan is also facing this situation, this paper aims to examine the rising incidence through transitions in nutrition and other lifestyle factors. Japan presents an interesting case because its population maintains the longest life expectancy in the world while also facing the consequences of rapid westernization and development [2]. The purpose of this review is to analyze the nutritional transitions and evaluate the effectiveness of current public health strategies in addressing key lifestyle diseases in Japan. Proposals for public health policies based on existing models in other countries are also given as actionable recommendations, and how they can be fit within a Japanese context.

**Figure 1:**
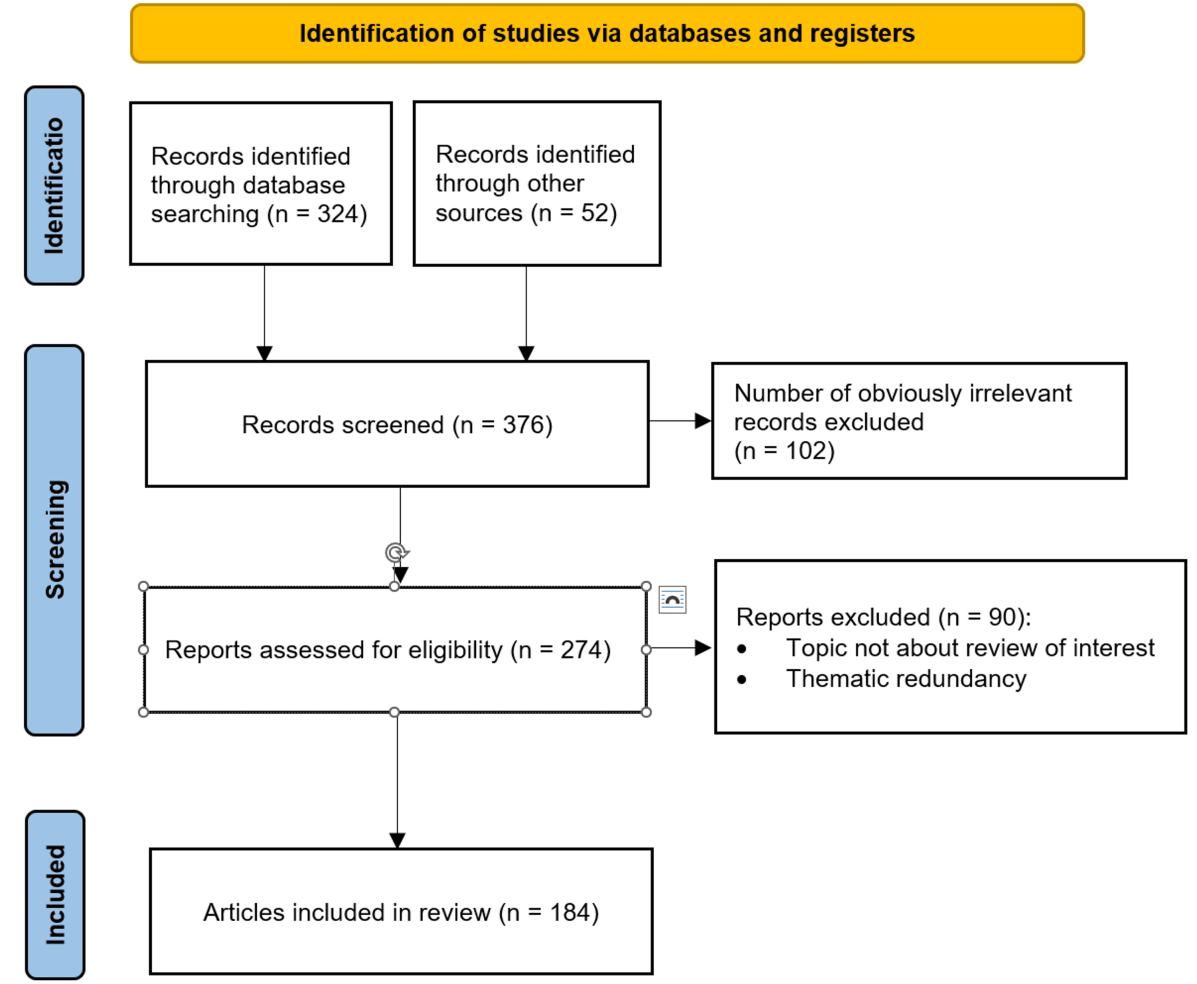
A flowchart of the review methodology

Data used for this review included searches conducted in academic scientific databases, including PubMed, Google Scholar, and data from the World Health Organization (WHO), the Statistics Bureau of Japan, and the Ministry of Health, Labor, and Welfare (MHLW). Inclusion criteria included references that are indexed, a government resource, peer-reviewed, or in the reference section of analyzed studies. Studies were excluded if they were not in English, Japanese, or a Nordic language; not related to lifestyle factors, non-lifestyle diseases (e.g., cancers), or acute diseases (e.g., stroke); or not about the Japanese population living in Japan. A preliminary, manual search was performed using the following terms: “Japan,” “non-communicable diseases,” “chronic diseases,” “lifestyle,” “diet,” “nutrition transition,” “climates,” “demographics; social, economic, and health.” “Hypertension” and “obesity” were determined to be notable non-communicable diseases (NCDs), and another search using these terms was performed in combination with the previous terms or the following: “pathophysiology,” “risk factor,” “epidemiology,” “prevalence,” and “trend.” To establish evidence-based recommendations, the American College of Lifestyle Medicine (ACLM) was sourced [3], and a final search was performed using additional terms: “government,” “policy,” “laws,” “regulation,” “sodium,” “alcohol,” “physical activity,” and “food labeling.” Figure 1 illustrates the flowchart of the review methodology.

## Review

Geography, economy, and agriculture

Japan is an archipelago located west of the Pacific Ocean in the Northern Hemisphere. It spans thousands of islands, with the four main islands being Hokkaido, Honshu (the "mainland"), Shikoku, and Kyushu, with a total reported population of 125,284,630 [4].

Japan is a high-income country with a highly diversified, trade-oriented economy and has a gross domestic product (GDP) per capita of an estimated $34,366 [4]. The main industries are automobiles, consumer electronics, computers, and other electronics. The largest source of employment is the service sector at approximately 73%, followed by industry at approximately 24% and agriculture at just 3% [5]. In addition to a small population currently working in agriculture, the number is further declining due to an aging population and fewer individuals entering the industry, so Japan relies on importing food to meet demand [6].

The islands experience four distinct seasons with a climate ranging from subarctic in the north to subtropical in the south. Its geography largely consists of expansive mountainous regions with flatter areas near the coast [7]. Agricultural land is also relatively scarce at just 12.72% of total land, with much of the farming done on slopes [8]. Japan also has a large coastline and exclusive economic zone, which contributes to productive fishing grounds [9]. The islands are susceptible to natural disasters such as earthquakes and typhoons, which can reduce yield and deal substantial damage to infrastructure. The top agricultural products grown are rice, milk, sugar beets, vegetables, eggs, chicken, potatoes, cabbage, sugarcane, and pork [8]. Given access to agriculture, the traditional Japanese diet was largely plant- and seafood-based, which included soybean-derived products (e.g., tofu and soy sauce), fish, fruits and vegetables, fermented or pickled foods, rice, and miso soup [10]. Due to the history of Buddhism and government policies, consumption of animal fat and meat was traditionally lower and not as pervasive in Japanese culture [11]. Currently, many areas, especially urban cities, have easy access to convenience stores, which contain many options for processed foods, such as snacks and prepackaged meals.

Nutrition transition and physical activity

Following the events of the Second World War, Japan began to develop economically with influence from Western countries, which also marked a shift in diet and nutrition [12]. This shift led to a decrease in Japan’s food self-sufficiency rate, which is defined as the percentage of food consumed that is produced domestically. In terms of calories, the food self-sufficiency rate has decreased from 79% in 1960 to 38% in 2022 [13], indicating a rise in the consumption of imported foods. For example, amounts of animal products and total fats and oils increased by four- and threefold, and consumption of traditional staples such as rice and fish has decreased [14]. The increase in imports of meat, oils, and sugars has raised the percentage of energy derived from fats despite the average total calorie intake staying roughly the same [14]. And as workers move away from agriculture towards other industries, such as the service sector, more of their working hours are spent sitting. Combined with a decrease in leisure-time physical activity, the decrease in manual labor has contributed to a more sedentary lifestyle [15]. Rapid changes in both diet and physical activity levels contribute to an increased rate of chronic diseases [16].

Chronic disease rates

**Figure 2:**
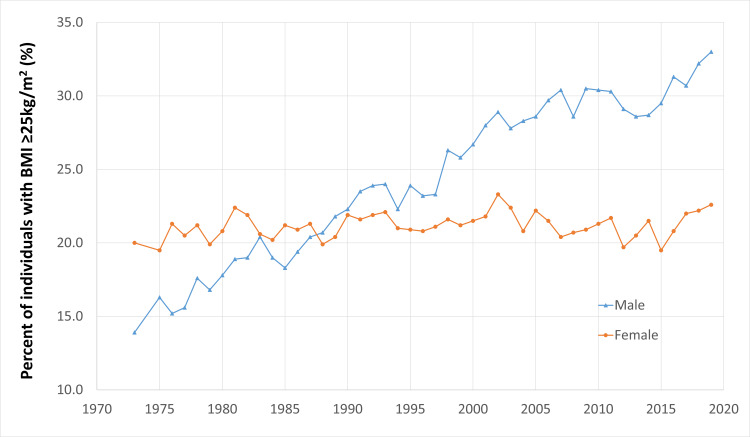
Percentage of males and females (excluding pregnant and lactating individuals) age 20 and above with obesity This figure has been created by the authors based on data from the Ministry of Health, Labour and Welfare's (MHLW) National Health and Nutrition Survey [21,22].

In Japan, an estimated 84.8% of deaths can be attributed to NCDs, and contributing risk factors include high blood pressure, tobacco smoking, alcohol consumption, and obesity [17]. Although systolic blood pressure has been decreasing in the general population, diastolic blood pressure in men has not shown a clear decreasing trend over the past 50 years [18]. In addition, the combined treatment and control rates indicate that only 15% to 30% of all hypertensive people were controlling their blood pressure at less than 140/90 mmHg. As a point of comparison, the prevalence of hypertension in Japan is approximately 31.4% and is very similar to that of the United States, which is 31.6% [17, 19]. While Japan has one of the lowest obesity rates in the world at 5.54%, increased weight is an ongoing concern [17]. Compared to populations in Western countries, Japanese individuals tend to develop obesity-related diseases at a lower body mass index (BMI), so the Japan Society for the Study of Obesity (JASSO) defines obesity at a BMI of ≥25 kg/m² [20]. Using this definition, the percentage of obese individuals in Japan is approximately 25%, which is more in line with other European countries. However, it is still less than the United States, which sits at 42% [19]. There has also been little change in the percentage of both males and females who are obese over the past decade (Figure 2) [21, 22]. The rest of this paper will use JASSO’s definition. These chronic diseases, like many NCDs, are largely preventable through lifestyle changes. There is sufficient evidence demonstrating the impact of diet, physical activity, and environmental exposures on the human body.

Nutrition and pathophysiology of hypertension and obesity

Sodium and Potassium’s Effect on Blood Pressure

There are many factors contributing to the pathogenesis of hypertension. Two frameworks describe how salt can cause hypertension: increased sodium intake activates the renin-angiotensin-aldosterone (RAA) system and impairs the activity of nitric oxide (NO), a vasodilator released from the endothelium. Although the kidneys have an important role in regulating sodium levels and plasma volume through mechanisms, sodium loading increases the activity of the brain RAA system, retaining more salt and water, thereby increasing blood pressure, and sodium accumulates in extracellular spaces that are more difficult for the kidneys to regulate [23, 24]. Salt loading also downregulates renal and vascular NO synthase expression and upregulates the endogenous inhibitor of NO synthase, asymmetrical dimethylarginine, contributing to rising blood pressure [25, 26]. Dietary potassium also has effects on hypertension by reducing blood pressure through mechanisms such as improved natriuresis, reduced sympathetic nervous system activity, and decreased pressor response to noradrenaline and angiotensin II, all of which oppose the effects of excess sodium [27]. An increased sodium-to-potassium ratio has been shown to be a risk factor for mortality, so increasing potassium intake is just as important as reducing sodium intake [28].

State of Sodium Consumption in Japan

The Japanese Society of Hypertension Guidelines for the Management of Hypertension 2019 recommended the target level of dietary salt restriction in patients with hypertension as less than 6 g/day, but levels remain high at 9.7 g/day in 2022 [21]. Sodium intake is high due to its presence in many staple items: most (63%) dietary sources are soy sauce (20%), commercially processed fish/seafood (15%), salted soups (15%), and preserved vegetables (13%) [29]. Urinary sodium excretion is a useful indicator of salt intake, and increased levels have been associated with Japanese pickles and miso [30]. Consumption of red meat is also associated with increased blood pressure and risk of heart disease because of increased sodium levels and saturated fat content [31]. Since the 1960s, meat and poultry consumption has increased dramatically in Japan, and fresh fish and shellfish have continued to decrease [32]. Fish is a core component of traditional Japanese meals and is very beneficial because it contains many bioactive components, such as omega-3 polyunsaturated fatty acids, which are protective against hypertension and other cardiovascular diseases [33]. As discussed above, potassium is also an important part of regulating blood pressure and is found in high quantities in foods such as fruits and vegetables, fish, and milk [34]. Although vegetable dishes are an integral part of the traditional diet, the quantity may not be sufficient to reach the recommended levels, and dietary patterns become more diverse and plant consumption decreases as Westernization of diet occurs [35]. Another factor contributing to undesirable sodium and potassium intake in Japan is the increased use of restaurant meals, takeout, and processed foods [36]. In 2019, approximately 41.6% of men and 26.7% of women ate out at least once per week [37]. Consumers of these foods typically do not have control over the seasoning, and consumption of meals away from home is associated with a lower intake of vegetables and potassium [38]. But due to convenience, these food sources are popular with Japanese workers who tend to work long hours and do not have the time to cook healthier meals at home [39]. Because of sodium’s ubiquity in the Japanese diet, continued excess intake and low vegetable consumption pose a threat to public health by increasing the risk of hypertension and associated chronic diseases in a large proportion of the population.

Alcohol’s Role in Blood Pressure

Another culprit of hypertension is alcohol. It can induce a biphasic effect on blood pressure: the first few hours after ingestion cause a decrease in blood pressure, and it increases after this period [40]. Sustained consumption in a dose-dependent manner also increases the risk of hypertension [41]. A possible mechanism is that alcohol consumption increases sympathetic nervous system activation and sympathetic amine release, both of which induce vasoconstriction and heart rate increase [42-44]. Alcohol may also stimulate the release of adrenaline, which increases heart rate, cardiac output, and systolic blood pressure [45]. Another possible mechanism is an imbalance of endogenous vasoconstrictors, such as endothelin-1 and angiotensin II, and vasodilators, such as NO [46]. Alcohol stimulates endothelin-1 and -2 release from the vascular endothelium in a dose-dependent manner [47]. Chronic alcohol ingestion also increases the levels of angiotensin II, which not only vasoconstricts but also stimulates NADPH oxidase activity in the vascular wall to create superoxide, causing endothelial damage [48-51]. NO is produced by endothelial nitric oxide synthase (eNOS) in the endothelium. Superoxide can reduce NO levels by reacting to form peroxynitrite radicals [52]. Alcohol can inhibit NO production and eNOS protein expression [53, 54]. These combined mechanisms can contribute to the impairment of the ability to vasodilate.

Alcohol in Japanese Society

**Figure 3:**
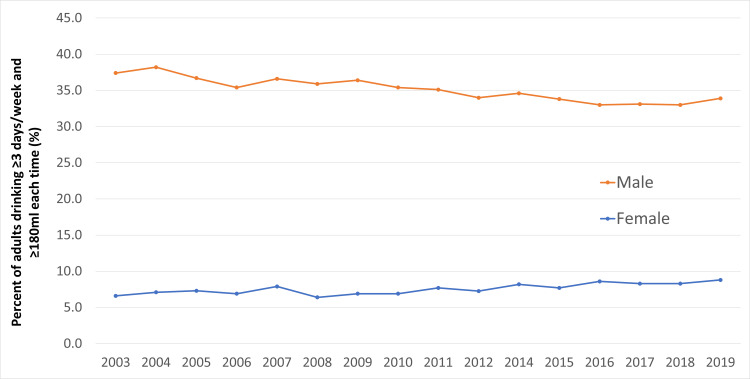
Percentage of adults aged 20 years and above who drink at least three times a week and at least 180 ml each time. This figure has been created by the authors based on data from the Ministry of Health, Labour and Welfare's (MHLW) National Health and Nutrition Survey [55].

After the Second World War, Japan's industrial and economic development increased wealth and alcohol availability, which diversified the drinking population of what was primarily just men and increased consumption overall [55]. Even now, alcohol is easily accessible in numerous establishments, such as restaurants and pubs, and sold in vending machines scattered around cities. Japanese drinking culture may also play a role in alcohol consumption, especially in men. While an average of 8.8% of women drink at least three times a week, the proportion is much higher in men, with an average of 33.9% in 2019, and the trend has not shown much change for either, as seen in Figure 3 [56]. In Japanese society, men and women lead gender-segregated lives even when they are married to each other, and the typical Japanese “salaryman” (a loyal, male, white-collar worker) spends much of his working life apart from his family [57]. After working long hours, they then gather after work with colleagues in bars for *enkai *(drinking meetings) to socialize and release stress. Alcohol consumption is more accepted in Japan than in the West because of the belief that "drinking facilitates socialization and mutual understanding between individuals" [58, 59], and this is very common amongst these salarymen who claim that drinking with clients or coworkers is part of their job [60]. Consequently, most of their social support is from work colleagues, which may be reinforcing heavier drinking behavior amongst each other and perpetuating the normalization of alcohol in one’s lifestyle. Despite alcohol’s declining popularity amongst the younger generation [56], its consumption is still a concern to be addressed in older individuals who are at greater risk of developing hypertension.

Sugar, Fat, and Obesity

Obesity is also another contributor to hypertension. A mechanism of obesity is desensitization of the body to leptin, a hormone that normally causes satiety by binding to the hypothalamus after release from adipocytes. However, excess adipose tissue increases leptin levels and induces hypothalamic resistance to this signal [61]. Additionally, consumed saturated animal fat also crosses the blood-brain barrier, causing direct damage to the hypothalamus and dysfunction of the appetite-regulating center [62, 63]. This indicates that increased consumption of saturated animal fats contributes to the development of obesity. In addition, it is believed that the modern diet already provides enough energy through carbohydrates and fats to meet the human daily energy requirements, so meat protein, which is digested slowly, contributes to an energy surplus and becomes stored as fat when eaten in excess [64,65].

Fructose is a simple sugar commonly found in many processed food products as a component of sucrose and high fructose corn syrup. Notably, it is a large part of sugary drinks, and Japan had the highest annual consumption among Asian countries in 2019 [66]. Approximately half of all sugar consumption in Japan is used for soft drinks, and increased demand for sugar is due to soft drinks [67]. When fructose is metabolized by the liver, it quickly consumes intracellular adenosine triphosphate (ATP) due to quick phosphorylation and will eventually lead to an increase in lipogenesis [68]. It also blocks fatty acid oxidation, further promoting liver fat content, which correlates with visceral fat [68, 69]. Visceral fat is an important measure because people of East Asian descent tend to accumulate more visceral fat compared with those of European ancestry, which may explain why the Japanese develop central obesity and obesity-related health disorders at a lower BMI [70-73]. Excessive visceral fat distribution is accompanied by hormonal, inflammatory, and endothelial alterations that induce stimulations of mechanisms that contribute to a hypertensive state [74]. According to microneurographic studies, sympathetic activation is greater in patients with visceral body fat distribution [75]. Increased sympathetic activity through obesity contributes to changes in levels of endothelin, angiotensin II, reactive oxygen species, and NO, causing increased blood pressure [74], the mechanisms of which are similar to alcohol consumption as discussed above. Increased sympathetic activity has also been found to increase renal tubular reabsorption of sodium in obese subjects, also contributing to increased blood pressure [76]. Increased consumption of fat and sugar contributes to rising obesity rates and may be due to the Westernization of diet and lifestyle [77].

Obesity in Japan

Convenience stores, vending machines, bars, and restaurants in Japan provide access to foods of varying degrees of healthiness. However, there is some evidence that increased usage of convenience stores is associated with poorer diet quality, such as decreased fruit and vegetable intake and increased carbohydrate intake [78]. Better access to fast food restaurants or convenience stores is also associated with higher BMI among those who live alone [79], and a study found that having many places nearby where children could buy processed foods or beverages was associated with more obesity [80]. Consumption of processed and fatty ready-to-eat foods was associated with decreased intake of healthier traditional Japanese ingredients such as fish and vegetables, and there may also be elevated liver enzymes indicating early stages of excessive liver fat accumulation [81]. Eating out also has been associated with greater mean daily energy intake [82], and increased fast food consumption is associated with obesity [83]. Salarymen are also susceptible to increased fat intake, leading to increased risk for obesity, as alcohol in drinking meetings is typically accompanied by fatty or other unhealthy foods [57]. Unlike alcohol, the public health impact of these foods is much greater, as people of all ages have easy access and are vulnerable because of their genetic predisposition.

The prevalence of high blood pressure is the most preventable risk factor for cardiovascular disease and total mortality [18], and it is a major risk factor for death from NCDs among adults in Japan [84]. Overweight and obesity were also associated with an increased risk of stroke and myocardial infarction [85]. Healthcare expenditure has been rising in Japan since 1995, and the demand for treatment and management of NCDs in a rapidly aging population will only exacerbate the issue in the coming years [86]. As such, it is important to educate the public on the impact of diet and lifestyle decisions through public health initiatives.

Recommendations: lifestyle medicine

Lifestyle diseases are a result of the gradual normalization of harmful lifestyle behaviors, which is what makes them difficult to address. These behaviors could be due to culture and customs or a recent and rapid development in society, both of which can conflict with recommended lifestyle modifications. However, the growing concern of modern lifestyle diseases in Japan can be largely attributed to Westernization. The traditional Japanese diet has been shown to promote good nutrition and health [87], and its cultural familiarity and role can be used to encourage lifestyle changes. Improving diet is a core part of lifestyle medicine and can be used to manage chronic diseases and reduce resulting death and disability. According to the ACLM, lifestyle medicine’s core principle is to use evidence-based therapeutic approaches, such as “whole-food, plant-predominant eating patterns, physical activity, restorative sleep, stress management, avoidance of risky substances, and positive social connections,” to treat and prevent chronic disease [3]. Unlike conventional medicine, which uses medications as the primary approach to treatment, lifestyle medicine aims to treat the underlying causes of diseases first and use medications second. Under lifestyle medicine, physicians should educate and involve patients in their own care and help them understand the consequences of risky choices [88]. Applying lifestyle medicine principles in Japan involves encouraging healthy eating, physical activity, and awareness and education of improved well-being.

The Japanese government has made steps towards targeting public health issues and reducing NCDs starting as far back as the late 20th century [89]. More recently, the Third National Health Promotion Measures, also known as Health Japan 21, was started in 2000 in response to the increasing prevalence of lifestyle-related diseases and their growing impact on an aging population [90]. Its goal was the primary prevention of NCDs, improvement of quality of life, and increasing the healthy life expectancy [89]. To do so, they gathered nationally representative data to analyze and target risk factors and diseases, and used that data to set concrete numerical targets for areas such as nutrition and diet, alcohol, and cardiovascular diseases such as hypertension. Obesity, salt reduction, and other factors were important for the prevention of hypertension, so the effectiveness of these lifestyle factors was also evaluated. Plans to improve these factors were enacted by both the national and local governments with the active participation and cooperation of various relevant organizations.

The promotion of *shokuiku *(“diet education” in Japanese) is important for bringing nutrition into more aspects of daily life, such as at home and school [91]. Initiatives like the National Health and Nutrition Survey were created to accumulate and organize scientific evidence for establishing the Dietary Reference Intakes (started in 2005 and revised in 2015) and the Dietary Guidelines for Japanese (started in 2000 and revised in 2016) [92]. The Japanese Food Guide spinning top was created to indicate recommended daily servings for each food group and encouraged a balanced diet of grains, fruits, and vegetables, protein, and milk [93], much like the United States’ MyPlate [94]. Basic dietary guidelines were promoted to educate the population, such as eating the traditional Japanese diet (*washoku*), avoiding excess salt consumption, and having regular meals [92]. Although *washoku *is not strictly plant-based, it incorporates more fish and less red meat than Western diets, and plant-based diets with limited animal products demonstrate lower blood pressures [95]. The second term of Health Japan 21 acquired data from 2010 to 2019, and despite creating and updating policies and initiatives, the final results did not show much improvement in some risk factors associated with NCDs, as seen in Table 1 [96]. As such, new targets were made for 2022, and data collection for the third term is underway.

**Table 1:** Risk factors associated with NCDs tracked in the second term of Health Japan 21 from 2010 to 2019. The MHLW used data from select years indicated in the table to act as a reference for setting the target values in 2022. BMI: body mass index; NCDs: non-communicable diseases This table has been created by the authors based on data from the Ministry of Health, Labour and Welfare (MHLW) National Health and Nutrition Surveys [96].

Indicator		Baseline	Results	Target for 2022
Average systolic blood pressure	Year	2010	2018	
	Males	138mmHg	137mmHg	134mmHg
	Females	133mmHg	131mmHg	129mmHg
Number of people with or at risk of metabolic syndrome	Year	2008	2019	25% reduction from 2008
	Number	14,000,000	15,160,000	
Obesity rates (BMI ≥25 kg/m^2^)	Year	2010	2019	
	Males ages 20-60	31.20%	35.10%	28%
	Females ages 40-60	22.20%	22.50%	19%
Average dietary salt intake	Year	2010	2019	
	Amount (grams)	10.6g	10.1g	8g
Percentage of people who exercise regularly	Year	2010	2019	
	Males aged 20-64 years	26.30%	23.50%	36%
	Females aged 20-64 years	22.90%	16.90%	33%
	Males aged ≥65 years	47.60%	41.90%	58%
	Females aged ≥65 years	37.60%	33.90%	48%
Percentage of people with increased risk of NCDs due to excess alcohol consumption (≥40g per day for men and ≥20g per day for women)	Year	2010	2019	
	Males	15.30%	14.90%	13%
	Females	7.50%	9.10%	6.40%

**Figure 4:**
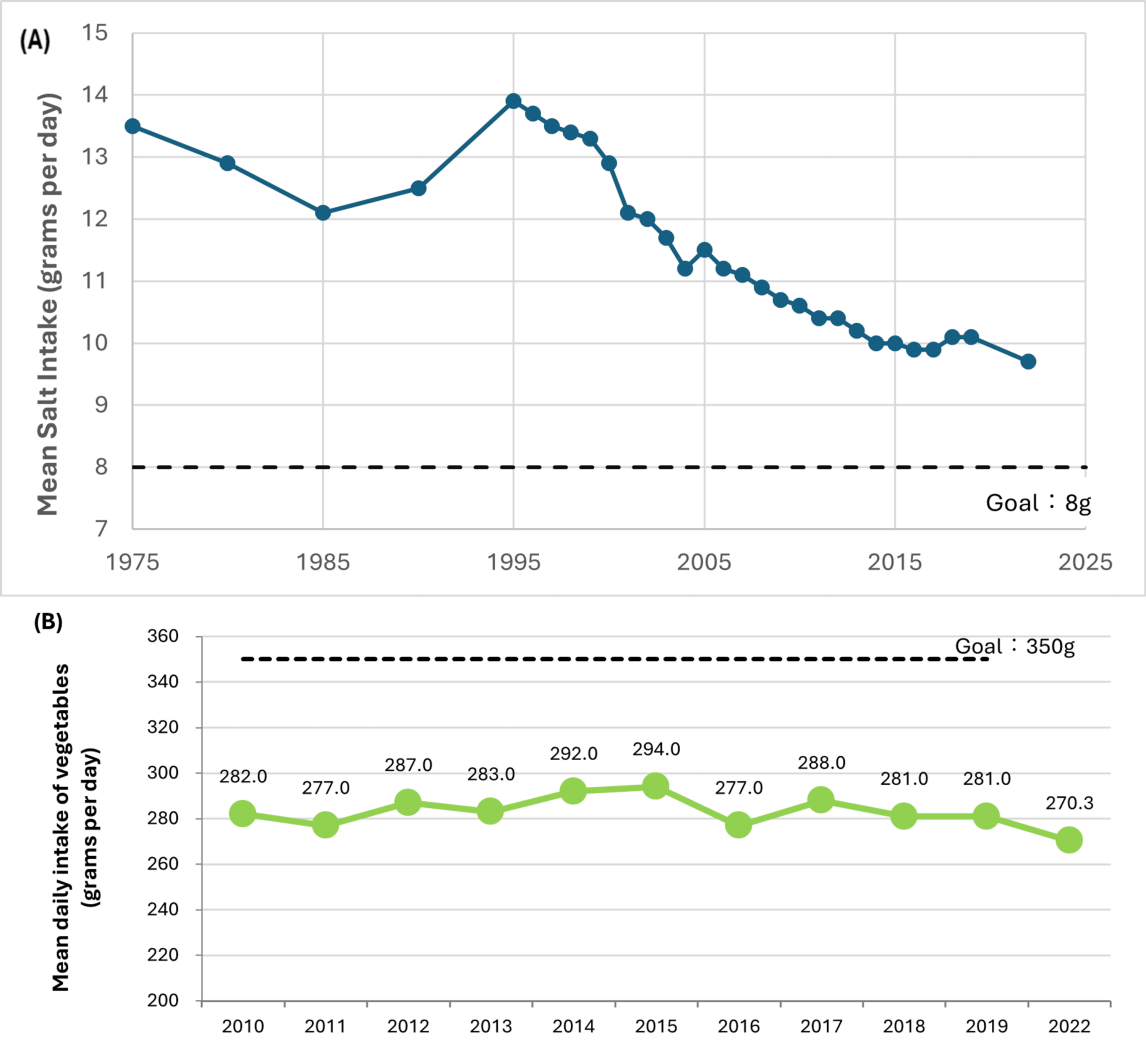
Changes in mean (A) salt and (B) vegetable intake of adults aged 20 years and above, with respective target levels as recommended by the MHLW. This figure has been created by the authors based on data from the Ministry of Health, Labour and Welfare's (MHLW) National Nutrition Survey for 1975-1990 and National Health and Nutrition Survey for 1995 to present [21,22,93].

The results could be explained by various factors. Diets continue to trend towards animal food over plant food and fish [35], and average intake of salt and vegetables has not reached the target recommended levels, as seen in Figures 4A , 4B [21, 97]. A growing number of people are prioritizing food purchases based on cost and convenience [98]. For example, premade salad purchases in 2023 were three times as much as those in 1986, and on the other hand, fresh vegetable purchases have decreased by approximately 23% in the same period. This can be due to an increasing general preference for westernized diets and lifestyles in Japanese society, as well as an increased availability of cooked and processed foods taking their place. In addition, poor weather and the decreasing number of farmers and amount of farmland over time have caused vegetable prices to increase and become more unaffordable. It has been found that those in the lower income bracket tend to consume fewer vegetables, so a rise in prices would only exacerbate this problem [99]. And while policies such as sodium reduction are beneficial for public health, enforcing them is challenging. *Shokuiku *encourages reducing salt usage in soups and dishes, which may be difficult due to traditional cooking methods. In response, there have been community volunteers holding classes to train parents and children on healthier recipes using less salt [92], and more food companies are supplying products low in salt and fat [89]. However, it can be difficult to change cooking habits deeply ingrained in one’s culture, and there may be pressure from family members not to alter familiar foods. Lowering the amount of sodium can cause flavor deficiency and texture deterioration, thereby decreasing enjoyment and possibly adherence to recommended guidelines. To improve public health and meet dietary goals, the government can consider more proactive measures to educate citizens about nutrition and make healthier choices.

Proposed Nutrient Taxes

Regulations such as enacting taxes on unhealthy foods and drinks can discourage consumption and direct diets towards healthier options. Sugar-sweetened beverage (SSB) taxes are a prominent example seen in many countries today [100]. They are associated with higher costs of taxed beverages and lower sales, which suggests consumers alter their purchasing habits in response to prices [101]. Although there has not been a sugar tax in Japan due to relatively less sugar consumption per capita compared to other high-income countries [102], it may need to be considered with the rise of SSB consumption. A salt tax may also be considered to combat high salt intake and hypertension prevalence. In 2011, Hungary passed a "Public Health Product" tax, which applied to many types of unhealthy foods, such as salty snacks and prepackaged sweets that exceeded the recommended threshold levels [103, 104]. In 2014, 16% of people who consumed salty snacks changed their behavior after the tax, but of these, only 5% chose healthier alternatives, whereas most either bought cheaper products or different brands [104]. While taxes did reduce the level of consumption of unhealthy foods, the impact was much greater for foods high in sugar than salt, possibly because of the lower base price of salt. There are currently very few real-world examples of salt taxes, and the few that exist, like in Hungary, involve taxing specific products such as salty snacks [103]. A possible system is taxing all foods based on salt content, which may be effective because foods with the highest levels are affected most, and it reduces options for cheaper substitutions. However, salt taxes face many challenges, which contribute to a lack of implementation. Unlike sugar taxes, which are narrower in scope by targeting specific products such as SSBs, salt is pervasive through many types of foods, and there is no singular "salty snack" to center around. There are also many different types of salts in food. The most common form is sodium chloride, also known as table salt, but potassium iodide exists as an alternative. Because sodium is a large component of elevated blood pressure, as discussed above, it may be effective to tax based on sodium content first. This provides an opportunity to gather data and allow manufacturers to change formulations to use less harmful alternatives. Salt is also an important part of many traditional Japanese foods, such as miso and salt-pickled dishes, so these taxes may cause public backlash and be culturally and politically difficult to implement. Lastly, the lack of real-world examples of salt taxes themselves is an obstacle to their introduction because there is less evidence of their effectiveness to build a case around. Despite this, food taxes targeting a broader range of salty foods should be considered, as modeling studies suggest they can be beneficial for reducing salt intake [103].

Nutrition Labeling

Strategies to educate and empower consumers to make healthier decisions, such as front-of-package labeling (FOPL), have been gaining popularity in many countries throughout the world. FOPL generally provide information on nutritional components, such as salt, fat, and sugars, and their quantities, but there is currently no global standard on formatting, so countries have varying presentations (e.g., size, shape, and color), public health nutrition messages (proscriptive, prescriptive, or both), and nutrient focus (e.g., “critical nutrients” only or including more positive and negative nutrients) [105]. In 2016, Chile passed the Law of Food Labeling and Advertising and became one of the few countries to have FOPL be mandatory by implementing black warning labels with "high in" symbols for products exceeding the limits of critical nutrients (sodium, fats, total sugars) and total energy (kilocalories). Impacts of this law included a significant decline in SSB purchases and in the proportion of foods available with high sodium or sugar levels due to reformulation by manufacturers [106-108]. This can prove to be a successful model for Japan, as vending machines showcasing a variety of SSBs are common throughout the country. If drinks have prominent labels indicating high levels of sugar, consumers can compare the available choices and make informed decisions at the time of purchase. Ready-to-eat meals can also benefit from aggressive food labeling. Since working adults have less time to cook for themselves, they frequently purchase these meals to meet their daily needs. Mandatory FOPL makes it easier to understand the general healthiness of a product and discourages purchasing meals with high salt levels or low potassium levels, for example. In response, Japanese manufacturers will need to reformulate their products to target the more health-conscious consumer.

Progress is already being made in Japan as of 2021, where at least 117 food companies registered with the MHLW's "Smart Life Project" and have initiatives dedicated to reducing salt and fat content in their foods [109]. Since the government and food companies have this existing relationship, it is possible for them to cooperate and create a labeling system that is most effective for consumers. There have been smaller-scale experimental studies demonstrating a reduction in purchases of foods with FOPL [110-112], but others showed no significant decrease [113, 114]. There are a few possible factors that may limit the effectiveness of FOPL. Firstly, the consumer should be able to understand and correctly interpret the nutrition information presented [115]. While the traffic light system is convenient by providing information at a glance with color coding, Nyilasy et al. [116] show that color labels on the nutrition facts panel may bias perception and cause healthy foods to be seen as less healthy even when the factual information is identical. Another issue is that knowing and understanding more information does not necessarily help consumers make healthier choices due to preexisting biases [117]. Tastiness is an important factor in determining food choice, and emphasizing the healthiness of a food with FOPL actually reduces perceived tastiness [117, 118]. This supports the "unhealthy = tasty" intuition and that having more nutritional information may not lead to healthier choices [118, 119]. Another barrier to a successful FOPL system is that these systems are still largely voluntary, where each manufacturer and retailer can apply an FOPL design freely or choose not to participate at all [115]. Multiple different FOPL designs can cause confusion when there is no common element, and the additional effort needed to decipher them would be a deterrent in real-life situations [120]. The Japanese government can address these challenges by supporting a single system based on its citizens' needs and strongly encouraging its adoption by all manufacturers and retailers for consistency.

Alcohol Regulation

**Figure 5:**
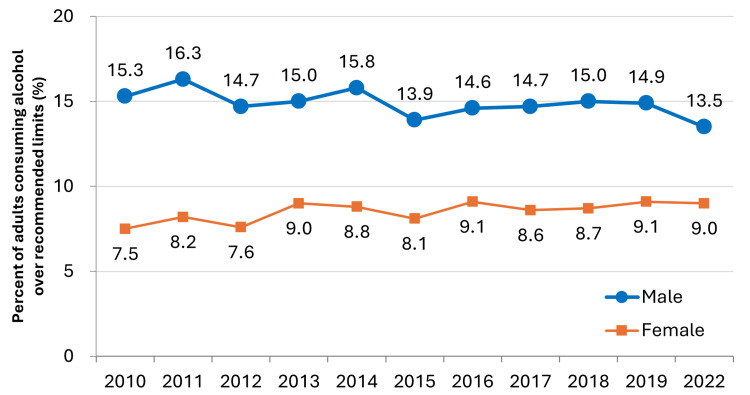
Percentage of adults aged 20 years and above who drink over the recommended limits (>40 g/day for males and >20 g/day for females). The target percentage for males and females is ≤13% and ≤6.4%, respectively. This figure has been created by the authors based on data from the Ministry of Health, Labour and Welfare's (MHLW) 2022 National Health and Nutrition Survey [21].

Data on Japan from the WHO shows there has been a slight decrease in total alcohol per capita consumption from 7.6 liters in 2000 to 6.3 liters in 2022 [121]. Figure 5 shows the trend of the percentage of people who drink over the recommended limits and the closing male-female gap in alcohol use [21]. Traditionally, it was middle-aged and older men who drank in Japan, but changes in social norms and gender role traditionality have diversified drinkers to include more women [122, 123]. The peak estimated drinking prevalence occurs during the 40s-50s, and age-standardized prevalence has been shown to continuously decrease for men in the past 20 years [124]. A factor could be that the population of young people is shrinking, so simply fewer people are drinking. Another factor could be due to changing social and cultural norms, as new policies with increased regulation, health promotion interventions, and attitude changes in social environments shape the perception of alcohol [125]. Alcohol and its consequences can appear less appealing, which pushes the younger generation away from drinking culture. The WHO found the most cost-effective methods to reduce harmful alcohol use include “increasing taxes on alcoholic beverages” and “enforcing restrictions on exposure to alcohol advertising” [126]. In Japan, there are several liquor taxes depending on the category it falls under, such as beer or wine, malt percentage in beers, etc. The national liquor tax reform was revised in 2017, and the tax rates for beer and wine will change in stages from 2020 to 2026 [127]. The goal of this tax reform was to consolidate the many categories into fewer, but in doing so, the tax rate for each category was either increased or decreased to reach the new target. It is known that increasing alcohol price is an effective method for reducing drinking [128], so this new reform may instead drive consumers towards alcoholic beverages that become cheaper rather than decreasing overall drinking.

**Figure 6:**
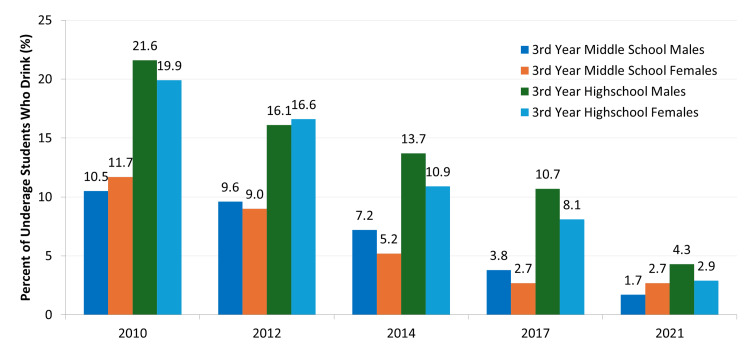
Percentage of students in the third year of middle school and high school who drank within the past 30 days of answering the survey. The target level for all groups is 0%. This figure has been created by the authors based on data from the Ministry of Health, Labour and Welfare's (MHLW) 2022 National Health and Nutrition Survey [22].

Another method of reducing consumption is targeting alcohol advertisements. In 2014, Japan passed the “Basic Act on Measures against Alcohol-related Harm,” and one of the goals included changing how alcohol is advertised to reduce underage drinking [129]. Although it has been on the decline, the incidence of minors drinking is still above the target level of 0% (Figure 6) [130]. At the time of writing, Japan does not have any restrictions for alcoholic beverage advertising in any form of media but instead relies on self-regulation through voluntary standards created by the Japanese alcohol industry [55, 131]. According to these guidelines, alcohol advertisements should not be aired from 5 AM to 6 PM, but the time period between 6 PM and 11 PM is when Japanese adolescents are most likely to watch TV and have been found to be exposed to two to 3.2 times more alcohol advertisements than at any other time of the day [132]. An association has also been found between exposure to alcohol advertising and current drinking among adolescents in Japan, indicating the importance of enacting regulations [133]. It was reported that a complete ban on alcohol advertising reduced recorded alcohol sales, suggesting its protective effect by reducing total alcohol consumption [134]. Restricting alcohol-promoting advertising and utilizing alcohol-warning advertising may also reduce consumption among heavy-drinking young adults [135], and advertising restrictions are inversely associated with the prevalence of hazardous drinking in people aged between 50 and 64 years [136]. Rather than having the industry continue to self-regulate, the government can take a stronger stance on advertising to eliminate underage drinking completely and reduce drinking in adults.

Lifestyle Education and Expanding Avenues for Healthcare

**Figure 7:**
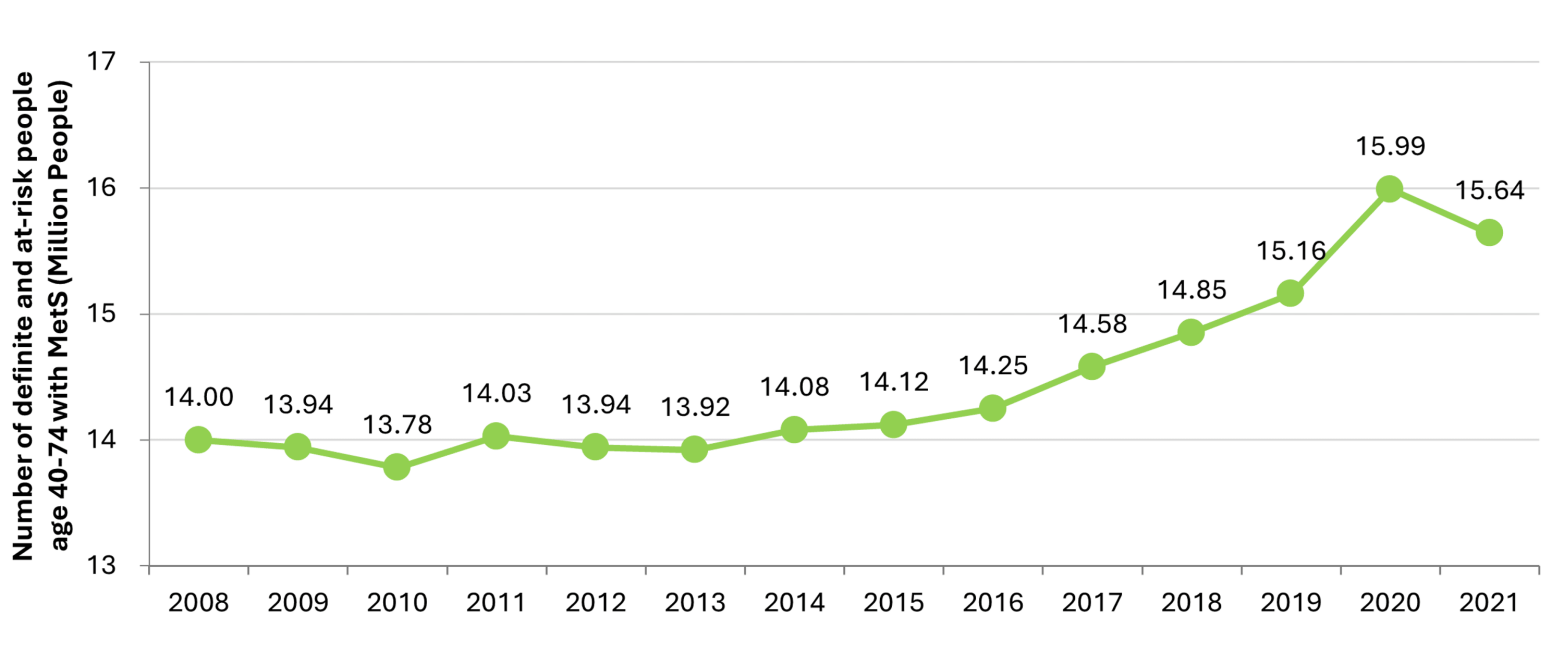
Number of people between the ages of 40 and 74 years who have definite MetS or are at risk of it. The national goal is to reduce the 2008 figure by 25%. MetS: metabolic syndrome. This figure has been created by the authors based on data from the Ministry of Health, Labour and Welfare's (MHLW) 2022 National Health and Nutrition Survey [21].

**Figure 8:**
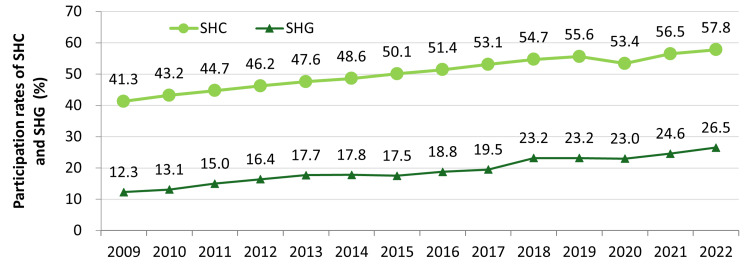
Percentage of people between the ages of 40 and 74 years who participate in SHC and SHG. The target levels for SHC and SHG are≥70% and ≥45%, respectively. SHC: Specific Health Checkups; SHG: Specific Health Guidance. This figure has been created by the authors based on data from the Ministry of Health, Labour and Welfare's (MHLW) 2022 National Health and Nutrition Survey [21].

The Japanese government also created the Specific Health Checkups (SHC) and Specific Health Guidance (SHG) programs in 2008 to incorporate lifestyle interventions as a part of NCD prevention with a focus on targeting metabolic syndrome (MetS) [137]. Figure 7 shows the rising number of people in Japan who are at risk or have MetS. SHC provides all insured individuals between the ages of 40 and 75 a yearly checkup, including, but not limited to, a lifestyle questionnaire (e.g., medications, smoking history), waist circumference, blood pressure, lipid panel, liver function, and blood glucose levels [138]. Based on the results, patients were sent educational material on how to improve their lifestyle, but those determined to have a high risk of NCDs were also referred to SHG, where they met one-on-one with their doctor and specialists (e.g., public health nurse or registered dietician) to get more detailed advice and support. Studies on the effectiveness of SHC and SHG have been conflicting, with some suggesting improvements on risk factors [137,139] and others not [140,141]. Figure 8 shows an increase in participation in SHC and SHG, respectively, but still falls below the target.

**Figure 9:**
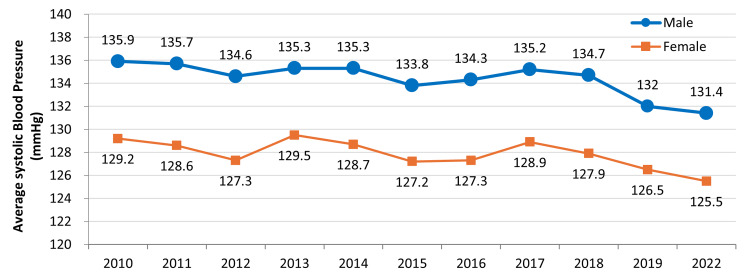
Average systolic blood pressure of males and females aged 20 years and above. Target pressures for males and females are 134 mmHg and 129 mmHg, respectively. This figure has been created by the authors based on data from the Ministry of Health, Labour and Welfare's (MHLW) 2022 National Health and Nutrition Survey [21].

Despite this, other studies show that a multidisciplinary approach in lifestyle intervention can be effective for long-term improvement of MetS risk factors such as hypertension [142, 143]. Figure 9 shows the decreasing trend in average systolic blood pressure for males and females. The Japanese government can consider expanding the team by including community pharmacies to work with patients at a more local level. For the prevention of hypertension, community pharmacy-based screenings can be a useful tool to improve awareness of blood pressure status, provide counseling for blood pressure control, and help refer those at risk to general practitioners for further evaluation [144, 145]. However, a single screening may increase unnecessary referrals, so two-day screening programs for hypertension may save individual and healthcare resources [146]. An example of second screenings is home blood pressure monitoring (HBPM), which offers several advantages over in-office measurements. HBPM has been found to give lower readings and is closer to the average blood pressure recorded during 24-hour ambulatory blood pressure monitoring [147]. HBPM can also be performed more often, provides more reproducible readings than office readings, and has improved correlations with measures of target organ damage [148-152]. HBPM can also avoid phenomena such as the white-coat effect, defined as elevated office blood pressure but low home blood pressure, or masked hypertension, defined as normal clinic blood pressure but elevated out-of-clinic blood pressure. Either condition can lead to inappropriate treatment of a patient if providers only rely on office blood pressure. HBPM adoption could be more important among Japanese people, as excess sodium intake may play a part in the genesis of masked hypertension [153]. By combining HBPM with input from a healthcare professional, such as through telemonitoring, treatment guidance can be given more quickly, effectively, and conveniently in response to readings [154-158].

However, there are challenges to incorporating HBPM for patients. For example, patients may have difficulty following the protocol and making it a part of their daily routine, increased anxiety from elevated blood pressure readings, or concerns about the accuracy, or financial difficulties purchasing a home blood pressure device [159]. The first two can be addressed by providing formal HBPM training and easily accessible clinical support through phone calls or in-person visits with physicians or community health professionals (e.g., pharmacists, nurses). To address cost, there are currently rental services in Japan, but they can become prohibitively expensive if used long-term. Instead, the government could support wider adoption by covering home blood pressure devices under insurance and providing them to patients directly or renting them at a low cost. Providers should also be trained on how to integrate HBPM into their care, as the 2019 Japanese Society of Hypertension guidelines strongly encourage the use of HBPM and have written extensive recommendations on implementation [160]. However, Japan is facing an aging society, so there will be fewer healthcare providers available relative to patients to provide vital lifestyle and prevention assistance [161]. Japan also has a public medical insurance system, so more people using healthcare services leads to greater expenses, and there will be fewer funds gained from taxes directed towards healthcare as more citizens go into retirement. However, providing resources for prevention and screening of NCDs can improve the long-term health of patients, thereby reducing the likelihood of more serious, resource-demanding conditions.

Physical Activity

**Figure 10:**
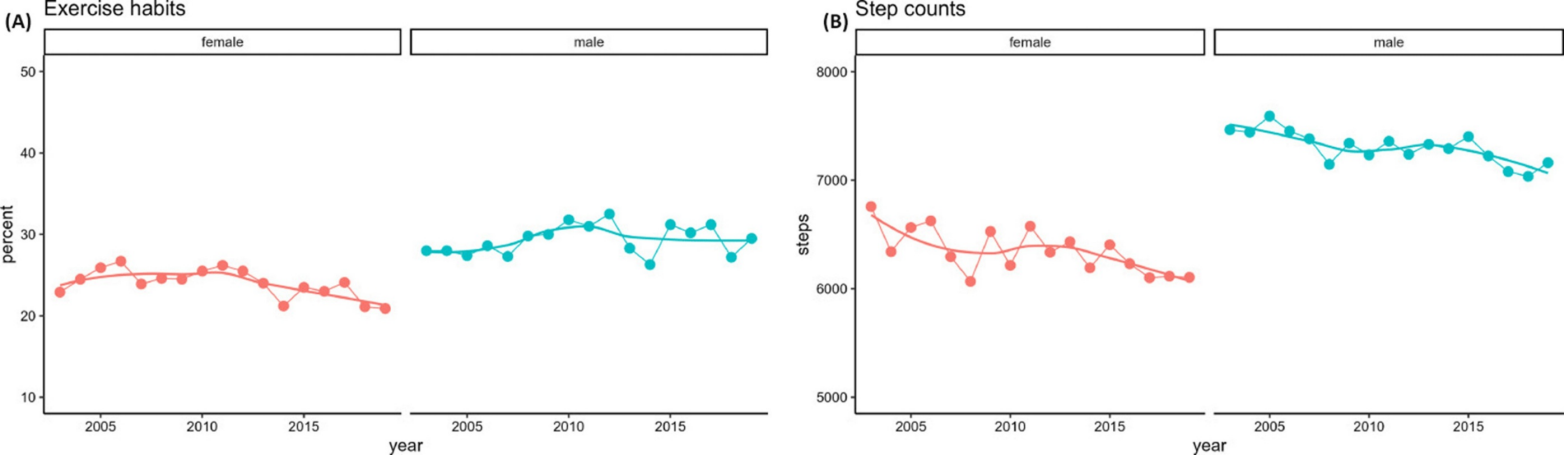
(A) Trends in the age-adjusted prevalence of exercise habits by gender from 2003 to 2019 and (B) trends in the age-adjusted pedometer-measured step counts by gender from 2003 to 2019. This image has been reproduced from Nakagata T, Ono R. (2024). Data resource profile: exercise habits, step counts, and sedentary behavior from the National Health and Nutrition Survey in Japan. Data Brief, 53, 110103. https://doi.org/10.1016/j.dib.2024.110103. Licensed under CC BY 4.0 (http://creativecommons.org/licenses/by/4.0/) [168].

In addition to more frequent checkups and education, the Japanese government started initiatives to increase physical activity in its population. In 2013, the MHLW published guidelines based on a systematic review of literature discussing the influence of physical activity on mortality and the onset of NCDs [162]. The main message of the guidelines was "+10," standing for "an additional 10 minutes of physical activity per day." They encourage people to start by incorporating those 10 minutes in their daily life with brisk walks, stretches, or simple strength training while in the office or at home, such as squats [163]. The goal is to continue building up those +10 minutes and incorporate more vigorous exercise where possible until they reach, and hopefully maintain, at least 60 minutes per day if 18-64 years old and 40 minutes per day if 65 and above. Studies have shown that even a small amount of daily activity, such as five minutes of running or 15 minutes of walking, can positively influence cardiovascular mortality and increase life expectancy by three years [164,165], and a lifestyle-based intervention with brisk walking of at least 10 minutes can improve MetS in overweight males [166]. Despite its importance in improving obesity and cardiovascular risks, rates of regular exercise in Japanese people have not improved, especially among working-age people who cite a lack of time and overtime working [167]. The 2019 data show the prevalence of exercise habits (defined as exercise frequency of ≥2 times a week, duration of ≥30 minutes at one occasion, and continuation of ≥1 year) was 20.9% in females and 29.5% in males, which is a significant decrease in females and no significant change in males since 2013 (Figure 10A) [168]. Daily step counts in both men and women have also seen declines in the same period: an average of 7162 steps in males and 6,105 in females in 2019, which are still below the target of 8000 steps (Figure 10B) [168]. In addition, Japanese adults also spend an average of 5.3 hours/day in sedentary behavior, and 25.3% spend ≥8 hours/day [169]. Another study found that among 20 countries, Japan had the highest percentage of participants with sitting time ≥9 hours/day, but these participants were mostly <40 years old [170]. A possible reason is that Japanese workers spend approximately 70% of their working hours in sedentary behavior, compared to the average sitting time of 42.7% in the U.S. [171, 172].

As a lot of time spent during work is sedentary, promoting physical activity through workplace programs can be a good place to start. For example, Sweden established a voluntary national program called the “wellness allowance” in 1988 [173,174]. This is a tax-free benefit valued at no more than 5,000 Swedish Krona (approximately $520 USD) per year provided by the employer to employees for certain activities with elements of exercise or other wellness, such as a gym card, riding lessons, team sports, massages, etc. Employers can also offer a “nature benefit” where they directly provide or pay for activities such as access to a gym at the workplace or massage treatments at work. In addition to monetary benefits, many employers in the private sector and the government also allow staff to perform physical activities during regular work hours, usually around one hour per week, to promote a healthy lifestyle. While this program is completely voluntary for employers, taking part in it demonstrates investment in their staff and cultivates a positive culture surrounding health and wellness. However, there is currently little data analyzing the effect of the wellness allowance on exercise levels in employees, even though it has been in effect for nearly 40 years. Past surveys have shown that about 50% of employees are offered wellness allowances, and just 60% to 70% of these are using them, where the biggest barriers include lack of motivation and time [175, 176]. Despite this, Sweden’s population has high levels of physical activity: 64% to 67% of total 16-84-year-old individuals exercised for at least 30 minutes per day from 2004 to 2015 and at least 150 minutes per week from 2016 to 2024 [177]. This difference compared to Japan may be attributed to better work-life balance, providing more leisure time for exercise, and the Swedish culture’s emphasis on staying healthy and active. While all aspects of Sweden’s model can be difficult to implement in Japan, other research has shown that providing fitness programs for office workers [178] and allowing exercise during work hours [179] improve physical activity levels and reduce sedentary time.

One such program is the practice of booster breaks, defined as "organized, routine work breaks intended to improve physical and psychological satisfaction and sustain or increase work productivity" [180]. The breaks are a group-based activity led by trained coworkers and can be done without changing clothes. Sessions are approximately 15 minutes each, consisting of four phases: warm-up (aerobic movements for one to two minutes), aerobic/toning/strengthening/stretching movements (10 to 12 minutes), cool-down (flexibility movements for one to two minutes), and relaxation/visualization (15 to 30 seconds). By implementing booster breaks throughout the workday, employees can break long stretches of sedentary time and increase physical activity without concerns about lack of time or access to equipment. A small-scale feasibility test showed improved high-density lipoprotein (HDL) cholesterol and weight loss, but there were only 14 participants in one small business, so the results are not generalizable [181]. Another study had more participants, and consistent attendees to the program had increased weekly pedometer counts, decreased sedentary behavior, and maintained BMI status, while those who took regular breaks significantly increased their BMI, but limitations included predominantly female participants, so results may not be generalizable to men or those in other professions [182]. Other programs, such as Move to Improve, also include 10- to 15-minute breaks of physical activity, and results show an increase in moderate and vigorous physical activity and daily steps [183]. Despite potential health benefits of introducing physical activity breaks into the workplace, there are some barriers that could prevent their adoption, such as existing work culture and lack of support from management, schedule/time constraints, interruption of workflow, and lack of interest/motivation [184]. For physical activity breaks to be successful, organizations need to be supportive of employees by encouraging participation, and managers should also participate to foster unity. There should also be flexibility within the schedule, so breaks are not disruptive to workers' tasks and do not cause feelings of guilt or annoyance. The workplace should also provide lifestyle education alongside breaks to motivate employees and strengthen commitment to health changes. While there is still more research to be done on effectiveness and implementation in the Japanese workplace, booster breaks and similar programs are opportunities to add structured, low-cost, and easily accessible time for physical activity into an employee’s daily life.

## Conclusions

Japan is facing chronic noncommunicable diseases such as hypertension and obesity. The evolutions in diet discussed in this review are only a few of the many factors that demonstrate physiological connections between lifestyle decisions and public health. From this analysis, it is suggested that, in combination with prescribing lifestyle modifications, the government can address the growing health problems by creating policies aimed at regulating excess consumption and empowering the population with knowledge and opportunities to make positive changes. Actionable recommendations the government can take include collaborating with manufacturers to create healthier product formulations and with healthcare professionals to incorporate lifestyle medicine and increased blood pressure monitoring into their practice. They can also incentivize businesses to incorporate physical activity or wellness programs for working adults. While there have been successful models in other countries, future areas of research, such as conducting cost-benefit analysis for policies such as front-of-package labeling, alcohol regulation, and nutrient taxes for salt and sugar, will be necessary to adapt them to the needs of the Japanese population.

Incorporating successful changes at both the local and national levels will help prevent and reverse chronic diseases, so people live not only longer but also lead more fulfilling lives. Additionally, future research should explore the cost-effectiveness of these strategies, particularly front-of-package labeling, alcohol regulation, and nutrient taxes. Evaluating these measures through economic and behavioral lenses will be vital to ensuring that Japan’s public health policies are not only impactful but also scalable and sustainable over time.
